# Virological outcome among HIV infected patients transferred from pediatric care to adult units in Madrid, Spain (1997–2017)

**DOI:** 10.1038/s41598-020-70861-x

**Published:** 2020-10-09

**Authors:** Carolina Beltrán-Pavez, Miguel Gutiérrez-López, Marina Rubio-Garrido, Ana Valadés-Alcaraz, Luis Prieto, José Tomás Ramos, Santiago Jiménez De Ory, Marisa Navarro, Cristina Díez-Romero, Federico Pulido, Eulalia Valencia, África Holguín, The Madrid Cohort of HIV-Infected Children integrated in the Pediatric Branch of the Spanish National AIDS Network (CoRISpe), María José Mellado, Luis Escosa, Milagros García Hortelano, Talía Sainz, María Isabel González-Tomé, Pablo Rojo, Daniel Blázquez, Luis Prieto-Tato, Cristina Epalza, José Tomás Ramos, Sara Guillén, María Luisa Navarro, Jesús Saavedra, Mar Santos, Begoña Santiago, David Aguilera-Alonso, Santiago Jiménez De Ory, Itzíar Carrasco, Miguel Ángel Roa, María Penín, Jorge Martínez, Katie Badillo, Eider Oñate, Itziar Pocheville, Elisa Garrote, Elena Colino, Jorge Gómez Sirvent, Mónica Garzón, Vicente Román, Raquel Angulo, Olaf Neth, Lola Falcón, Pedro Terol, Juan Luis Santos, David Moreno, Francisco Lendínez, Estrella Peromingo, José Uberos, Beatriz Ruiz, Ana Grande, Francisco José Romero, Carlos Pérez, Miguel Lillo, Begoña Losada, Mercedes Herranz, Matilde Bustillo, Pilar Collado, José Antonio Couceiro, Leticia Vila, Consuelo Calviño, Ana Isabel Piqueras, Manuel Oltra, César Gavilán, Elena Montesinos, Marta Dapena, Cristina Álvarez, Beatriz Jiménez, Ana Gloria Andrés, Víctor Marugán, Carlos Ochoa, Santiago Alfayate, Ana Isabel Menasalvas, Yolanda Ruiz Del Prado, Pere Soler-Palacín, Marie Antoinette Frick, Antonio Mur, Nuria López, María Méndez, Lluís Mayol, Teresa Vallmanya, Olga Calavia, Lourdes García, María Teresa Coll, Valentí Pineda, Neus Rius, Joaquín Dueñas, Clàudia Fortuny, Antoni Noguera-Julián, Ignacio Bernardino, María Luisa Montes, Eulalia Valencia, Rafael Rubio, Federico Pulido, Otilia Bisbal, Gabriel Gaspar Alonso, Juan Berenguer, Cristina Díez, Teresa Aldamiz, Pedro Montilla, Elena Bermúdez, Maricela Valerio, José Sanz, Sari Arponen, Alejandra Gimeno, Miguel Cervero, Rafael Torres, Santiago Moreno, Mª Jesús Pérez, Pablo Ryan, Jesús Troya, Jesús Sanz, Juan Losa, Rafael Gómez, José Antonio Iribarren, Francisco Rodríguez, Lydia Pascual, María José Aramburu, Ane Josune Goikoetxea, Luis Aguirrebengoa, Josefa Muñoz, Sofía Ibarra, Michele Hernández, Juan Luis Gómez Sirvent, Jehovana Rodríguez, Miguel Ángel Cárdenes, Luis Fernando López-Cortés, Cristina Roca, Silvia Llaves, María José Ríos, Jesús Rodríguez, Virginia Palomo, Juan Pasquau, Coral García, José Hernández, Clara Martínez, Antonio Rivero, Ángela Camacho, Dolores Merino, Elisa Martínez, Fernando Mateos, José Javier Blanch, Miguel Torralba, Piedad Arazo, Gloria Samperiz, María José Crusells, Isabel San Joaquín, Celia Miralles, Antonio Ocampo, Guille Pousada, Álvaro Mena, Marta Montero, Miguel Salavert, Sandra Cuéllar, María José Galindo, Ramón Ferrando, Joaquín Portilla, Irene Portilla, Félix Gutiérrez, Mar Masiá, Cati Robledano, Araceli Adsuar, Carmen Hinojosa, Pablo Bachiller, Jésica Abadía, José Luis Mostaza, Rosario Pérez, Carlos Galera, Helena Albendín, Aurora Pérez, José Ramón Blanco, Joaquín Burgos, Berta Torres, Elisa Lazzari

**Affiliations:** 1 HIV-1 Molecular Epidemiology Laboratory, Microbiology and Parasitology Department, Hospital Ramón y Cajal-IRYCIS and CIBEREsp-RITIP-CoRISPe, Carretera de Colmenar Viejo, Km. 9,100. -2D, 28034 Madrid, Spain; 2 Department of Infectious Diseases, Hospital 12 de Octubre, RIS, Madrid, Spain; 3 Department of Infectious Diseases, Hospital Clínico Universitario and Universidad Complutense-CoRISpe, Madrid, Spain; 4 Hospital General Universitario Gregorio Marañón, Instituto de Investigación Sanitaria Gregorio Marañón (IisGM), CoRISpe, Madrid, Spain; 5 Department of Infectious Diseases, Hospital Gregorio Marañón, RIS, Madrid, Spain; 6 Hospital Universitario 12 de Octubre, imas12, UCM, Madrid, Spain; 7 HIV Unit, Internal Medicine Service, Hospital Universitario La Paz-IdiPAZ, Madrid, Spain; 8 Hospital Universitario La Paz, Madrid, Spain; 9 Hospital Universitario Doce de Octubre, Madrid, Spain; 10 Hospital Clínico San Carlos, Madrid, Spain; 11 Hospital Universitario de Getafe, Madrid, Spain; 12 Hospital Universitario Gregorio Marañón, Madrid, Spain; 13 Hospital Universitario de Móstoles, Madrid, Spain; 14 Hospital Universitario Príncipe de Asturias de Alcalá de Henares, Madrid, Spain; 15 Hospital Infantil Universitario Niño Jesús, Madrid, Spain; 16 Hospital Universitario de Torrejón, Madrid, Spain; 17 Hospital Universitario Donostia, Guipúzcoa, Spain; 18 Hospital Universitario Cruces, Vizcaya, Spain; 19 Hospital Universitario Basurto, Vizcaya, Spain; 20 Hospital Insular Materno Infantil, Gran Canaria, Spain; 21 Hospital Universitario Virgen de la Candelaria, Tenerife, Spain; 22 Hospital General, Lanzarote, Spain; 23 Hospital de Poniente de El Ejido, Almería, Spain; 24 Hospital Universitario Virgen del Rocío, Sevilla, Spain; 25 Hospital Universitario Virgen de la Macarena, Sevilla, Spain; 26 Hospital Universitario Virgen de las Nieves, Granada, Spain; 27 Hospital Regional Universitario Carlos Haya, Málaga, Spain; 28 Complejo Hospitalario Torrecárdenas, Almería, Spain; 29 Hospital Universitario Puerta del Mar, Cádiz, Spain; 30 Hospital Clínico San Cecilio, Granada, Spain; 31 Hospital Universitario Reina Sofía, Córdoba, Spain; 32 Complejo Hospitalario Universitario Infanta Cristina, Badajoz, Spain; 33 Complejo Hospitalario, Cáceres, Spain; 34 Hospital de Cabueñes, Asturias, Spain; 35 Complejo Hospitalario Universitario, Albacete, Spain; 36 Hospital Virgen de la Salud, Toledo, Spain; 37 Hospital Virgen del Camino, Navarra, Spain; 38 Hospital Universitario Miguel Servet, Zaragoza, Spain; 39 Hospital Clínico Universitario Lozano Blesa, Zaragoza, Spain; 40 Complejo Hospitalario Universitario, Pontevedra, Spain; 41 Complejo Hospitalario Universitario, La Coruña, Spain; 42 Hospital Universitario Lucus Augusti, Lugo, Spain; 43 Hospital Universitario La Fe, Valencia, Spain; 44 Hospital Universitario de San Juan de Alicante, Alicante, Spain; 45 Hospital General Universitario, Valencia, Spain; 46 Hospital General, Castellón, Spain; 47 Hospital Universitario Marqués de Valdecilla, Cantabria, Spain; 48 Complejo Hospitalario, León, Spain; 49 Complejo Hospitalario, Zamora, Spain; 50 Hospital Universitario Virgen de la Arrixaca, Murcia, Spain; 51 Complejo Hospitalario San Millán-San Pedro, La Rioja, Spain; 52 Hospital Universitari Materno Infantil Vall d’Hebron, Barcelona, Spain; 53 Hospital Universitari del Mar, Barcelona, Spain; 54 Hospital Universitari Germans Trias i Pujol, Barcelona, Spain; 55 Hospital Universitari Josep Trueta, Girona, Spain; 56 Hospital Universitari Arnau de Vilanova, Lleida, Spain; 57 Hospital Universitari Joan XXIII, Tarragona, Spain; 58 Consorci Sanitari del Maresme de Mataró, Barcelona, Spain; 59 Hospital General de Granollers, Barcelona, Spain; 60 Corporació Sanitària Parc Taulí de Sabadell, Barcelona, Spain; 61 Hospital Universitari Sant Joan de Reus, Tarragona, Spain; 62 Hospital Universitari Son Espases, Mallorca, Spain; 63 Hospital Sant Joan de Déu de Esplugues de Llobregat, Barcelona, Spain; 64 Hospital Universitario Severo Ochoa de Leganés, Madrid, Spain; 65 Hospital Universitario Ramón y Cajal, Madrid, Spain; 66 Hospital Universitario Infanta Leonor, Madrid, Spain; 67 Hospital Universitario La Princesa, Madrid, Spain; 68 Hospital Universitario Fundación Alcorcón, Madrid, Spain; 69 Hospital Universitario Insular, Gran Canaria, Spain; 70 Hospital Universitario de Canarias, Tenerife, Spain; 71 Hospital Universitario Doctor Negrín, Gran Canaria, Spain; 72 Hospital Universitario Clínico San Cecilio, Granada, Spain; 73 Hospital Universitario Juan Ramón Jiménez, Huelva, Spain; 74 Hospital Universitario, Guadalajara, Spain; 75 Hospital Álvaro Cunqueiro, Pontevedra, Spain; 76 Hospital Clínico Universitario, Valencia, Spain; 77 Hospital General Universitario, Alicante, Spain; 78 Hospital General Universitario de Elche, Alicante, Spain; 79 Hospital Clínico, Valladolid, Spain; 80 Hospital Universitario Río Hortega, Valladolid, Spain; 81 Hospital Universitari Vall d’Hebron, Barcelona, Spain; 82 Hospital Clinic, Barcelona, Spain

**Keywords:** Epidemiology, Outcomes research, Paediatric research, Infectious diseases, Clinical microbiology, Virology, Paediatrics

## Abstract

The aim of this transversal study was to describe the virological and immunological features of HIV-infected youths transferred from pediatric to adult care units since 1997 vs. the non-transferred patients from the Madrid Cohort of HIV-infected children and adolescents in Spain. We included 106 non-transferred and 184 transferred patients under clinical follow-up in 17 public hospitals in Madrid by the end of December 2017. Virological and immunological outcomes were compared in transferred vs. non-transferred patients. ART drug resistance mutations and HIV-variants were analyzed in all subjects with available resistance *pol* genotypes and/or genotypic resistance profiles. Among the study cohort, 133 (72.3%) of 184 transferred and 75 (70.7%) of 106 non-transferred patients had available resistance genotypes. Most (88.9%) of transferred had ART experience at sampling. A third (33.3%) had had a triple-class experience. Acquired drug resistance (ADR) prevalence was significantly higher in pretreated transferred than non-transferred patients (71.8% vs. 44%; p = 0.0009), mainly to NRTI (72.8% vs. 31.1%; p < 0.0001) and PI (29.1% vs. 12%; p = 0.0262). HIV-1 non-B variants were less frequent in transferred vs. non-transferred (6.9% vs. 32%; p < 0.0001). In conclusion, the frequent resistant genotypes found in transferred youths justifies the reinforcement of HIV resistance monitoring after the transition to avoid future therapeutic failures.

## Introduction

Globally, an estimated 1.7 million children below 15 years old were living with human immunodeficiency virus (HIV) by the end of 2018^1^. Young people (10–24 years), including adolescents (10–19 years), are vulnerable to HIV infection, mainly for adolescents who live in settings with a generalized HIV epidemic. In 2018, there were approximately 1.6 million adolescent people living with HIV^2^. The World Health Organization (WHO) estimates that one-seventh of all new HIV infections occur during adolescence.

Since 2005, HIV infection has become a chronic disease of childhood. Perinatally infected population live to adulthood and are transitioning from pediatric to adult care in an increasing number^3^. Perinatally infected children who reach adolescence have been exposed to various antiretroviral (ARV) drug regimens during their lifetime and have a higher risk of developing ARV resistance, compromising the success of present and future treatments options^4^. Indeed, adolescents living with perinatally acquired HIV and transferred to adult care units, have higher mortality^5^ and virological failure rates compared to younger children and adults^6, 7^. Thus, it is especially necessary to check clinical and virological status of this population, including periodic surveillance studies monitoring the drug resistance mutations (DRM) prevalence to the main ARV families in clinical use in order to ensure proper treatment^8^.

To date, few studies have investigated the clinical status and epidemiological data of transferred patients from pediatric to adult care. Among high-income countries, Spain has one of the most studied and well reported perinatal HIV cohort, with 1,335 HIV-infected children, adolescents and youths registered since 1995^9,10^. The present study updates the demographic, epidemiological and virological features by December 2017 in HIV-1 infected adolescents/youths transferred to adult units in Madrid with available resistance genotypes vs. patients under pediatric care.

## Results

### Baseline characteristics of transferred population in the Madrid pediatric Cohort

By the end of December 2017, 290 patients of the Madrid Cohort of HIV-infected children and adolescents were under clinical follow-up in 17 public hospitals in Madrid, Spain. A significant higher number of them were born in Spain vs. foreigners countries (84.1%, vs. 15.9%; p < 0.0001). A total of 279 had data related transmission route and 199 related to viral load, CD4 and CD8 counts. Among subjects with available data, a significant higher rate acquired the infection by vertical route (95.3%) vs. transfusion (2.9%) or sexual intercourse (1.8%). In the last available report, most presented ≤ 500 vs. > 500 HIV-1-RNA copies/ml (84.4% vs. 16.6%; p < 0.0001), > 350 vs. ≤ 350 CD4 cells/mm^3^ (89.4% vs. 10.6%; p < 0.0001). Most showed < 15% vs. ≥ 15% nadir CD4% (60.8% vs. 39.2%; p < 0.0001), < 200 vs. ≥ 200 nadir CD4 counts (41.2% vs. 58.8%; p < 0.0001), and ≥ 25% vs. < 25% CD8 rate (92.5% vs. 7.5%; p < 0.0001). Majority presented CD4/CD8 ratio < 1 vs. ≥ 1 (58.3% vs. 41.7%; p < 0.001).

Among them, 106 remained in pediatrics care units and 184 were transferred to adult care units from 1997 to December 2017. Table 1 summarizes their demographic characteristics. Both groups were mainly perinatally HIV-infected and 57% of them were female. By December of 2017 the mean age of the cohort was 27 (SD 4.2) years old for transferred and 15.6 (SD 5.7) years old for non-transferred patients. The median age at diagnosis was 1.3 (IQR 0.4–4.6) years for transferred and 0.6 (IQR 0.2–4.6) years for non-transferred patients. Most (90.7%) of transferred were diagnosed before the year 2000 and 74.4% in the 1990s. The transition of patients from pediatric units to adult health care occurred at median age of 18.7 (IQR 17.6–20.8) years old, and mainly (91.8%) after year 2002. The rate of transferred subjects with native Spaniard origin was significantly higher than in the non-transferred (92.9% vs. 68.9%; p < 0.0001). Only 6% of transferred youths were born in Africa or Latin America vs. 29.2% of non-transferred patients (Table 1). Considering the whole study cohort with available resistance data (n = 208), the rate of treated patients was significantly higher than those ARV-naïve (81.1% vs. 18.9%; p < 0.0001), as well as those with mono-dual vs. triple ARV class experience (52.3% vs. 25.9%; p < 0.0001).

**Table 1:** Demographic and virological-immunological features of non-transferred and transferred patients in the Madrid cohort at the end of December 2017.

Demographic characteristics	Non-transferred^a^ (N = 106)	Transferred^b^ (N = 184)	P value
**Female, No. (%)**	61 (57.5)	106 (57.6)	1.0000
**Route of infection, No. (%)**			
Perinatally	93 (87.7)	173 (94)	0.0766
Transfusion	2 (1.9)	6 (3.3)	0.7148
Sexual	2 (1.9)	3 (1.6)	1.0000
Unknown	9 (8.5)	2 (1.1)	**0.0024**
**Age, years, mean [SD], No. (%)**	15.6 [5.7]	27 [4.2]	**< 0.0001**
0 to < 6	7 (6.6)	0	**0.0008**
6 to < 12	22 (20.8)	0	**< 0.0001**
12 to < 18	40 (37.7)	3 (1.6)	**< 0.0001**
18 to < 24	31 (29.2)	44 (23.9)	0.3187
24 to ≤ 30	6 (5.7)	93 (50.6)	**< 0.0001**
> 30	0	44 (23.9)	**< 0.0001**
**Period of HIV diagnosis, No. (%)**			
1985–1989	0	30 (16.3)	**< 0.0001**
1990–1994	4 (3.8)	88 (47.8)	**< 0.0001**
1995–1999	23 (21.7)	49 (26.6)	0.3491
2000–2004	28 (26.4)	12 (6.5)	**< 0.0001**
2005–2009	20 (18.9)	1 (0.6)	**< 0.0001**
2010–2014	20 (18.9)	3 (1.6)	**< 0.0001**
2015–2016	10 (9.4)	1 (0.6)	**< 0.0001**
Unknown	1 (0.9)	0	0.3655
**Age at diagnosis, years, median [IQR], No. (%)**	0.6 [0.2–4.6]	1.3 [0.4–4.6]	0.0742
0 to < 6	86 (81.2)	149 (81.0)	1.0000
6 to < 12	14 (13.2)	27 (14.7)	0.8614
12 to ≤ 18	5 (4.7)	8 (4.3)	1.0000
Unknown	1 (0.9)	0	0.3655
**Calendar year of transfer, No. (%)**			
1997–1999	–	4 (2.2)	
2000–2002	–	9 (4.9)	
2003–2005	–	21 (11.4)	
2006–2008	–	29 (15.8)	
2009–2011	–	47 (25.5)	
2012–2014	–	42 (22.8)	
2015–2017	–	30 (16.3)	
Unknown	–	2 (1.1)	
**Age at transfer, years, median [IQR]**	–	18.7 [17.6–20.8]	
**Origin of birth**^**c**^**, No. (%)**			
Spain (West Europe)	73 (68.9)	171 (92.9)	**< 0.0001**
Portugal (West Europe)	0	1 (0.5)	1.0000
East Europe	0	1 (0.5)	1.0000
North Africa	2 (1.9)	2 (1.1)	0.6249
Sub-Saharan Africa	19 (17.9)	2 (1.1)	**< 0.0001**
South and Central America	10 (9.4)	7 (3.8)	**< 0.0001**
Asia	2 (1.9)	0	0.1328

Virological features^d^ No. (%)	Non-transferred N = 75 (70.7)	Transferred N = 133 (72.3)	P value
**Viral load, median [IQR], No. (%)**	35 [20–37]	37 [20–71]	**0.0441**
≤ 20	28 (37.3)	43 (32.3)	0.5427
21–50	25 (33.4)	44 (33.1)	1.0000
51–200	4 (5.3)	18 (13.5)	0.0983
201–500	1 (1.3)	5 (3.7)	0.4220
501–1,000	3 (4.0)	2 (1.5)	0.3536
1,001–10,000	3 (4.0)	9 (6.8)	0.5429
> 10,000	3 (4.0)	11 (8.3)	0.3876
Unknown	8 (10.7)	1 (0.8)	**0.0014**
**CD4 percentage, mean [SD], No. (%)**	33.3% [9.2]	31.6% [11.4]	0.2715
< 25%	11 (14.7)	35 (26.3)	0.0571
25–50%	55 (73.3)	90 (67.7)	0.4346
> 50%	1 (1.3)	7 (5.3)	0.2631
Unknown	8 (10.7)	1 (0.8)	0.2715
**CD4 cells/mm**^**3**^**, median [IQR], No. (%)**	781 [561–962]	725 [498–901]	0.279
≤ 200	2 (2.6)	7 (5.3)	0.4934
201–350	4 (5.3)	8 (6.0)	1.0000
351–500	8 (10.7)	19 (14.3)	0.5244
501–1,000	39 (52.0)	74 (55.7)	0.6646
1,001–1,500	11 (14.7)	22 (16.5)	0.8440
> 1,500	3 (4.0)	2 (1.5)	0.3536
Unknown	8 (10.7)	1 (0.8)	**0.0014**
**Nadir CD4 percentage, median [IQR], No. (%)**	15% [11–22.7]	11% [3–16.7]	**0.0001**
< 15%	33 (44.0)	88 (66.2)	**0.0022**
15–24%	22 (29.3)	36 (27.0)	0.7492
≥ 25%	12 (16.0)	8 (6.0)	**0.0265**
Unknown	8 (10.7)	1 (0.8)	**0.0014**
**Nadir CD4 (cells/mm**^**3**^**), median [IQR], No. (%)**	379 [206–500]	187 [41.2–345.5]	**< 0.0001**
< 200	15 (20.0)	67 (50.4)	**< 0.0001**
200–499	35 (46.6)	57 (42.8)	0.6632
≥ 500	17 (22.7)	8 (6.0)	**0.0007**
Unknown	8 (10.7)	1 (0.8)	**0.0014**
**CD8 percentage, median [IQR], No. (%)**	36% [28–42]	39.6% [34–53]	**0.0004**
< 25%	11 (14.6)	4 (3.0)	**0.0036**
25–50%	48 (64.0)	92 (69.2)	0.4466
> 50%	8 (10.7)	36 (27.0)	**0.0049**
Unknown	8 (10.7)	1 (0.8)	**0.0014**
**CD8 cells/mm**^**3**^**, median [IQR], No. (%)**	795 [552–1077]	873 [701–1210]	**0.0232**
≤ 200	1 (1.3)	1 (0.8)	1.0000
201–350	3 (4.0)	3 (2.2)	0.6694
351–500	7 (9.3)	7 (5.2)	0.2651
501–1,000	37 (49.4)	69 (51.9)	0.7735
1,001–1,500	16 (21.3)	37 (27.8)	0.3251
> 1,500	3 (4.0)	15 (11.3)	0.1208
Unknown	8 (10.7)	1 (0.8)	**0.0014**
**CD4/CD8 ratio [IQR], No. (%)**	0.9 [0.6–2.3]	0.8 [0.4–1.2]	**0.0205**
< 1	34 (45.3)	82 (61.6)	**0.0291**
≥ 1	33 (44.0)	50 (37.6)	0.0849
Unknown	8 (10.7)	1 (0.8)	**0.0014**

^a^Patients from Madrid Cohort of HIV-1 infected children and adolescents under follow-up in pediatric units.

^b^Transferred from pediatric to adult units.

^c^Birth origin of patients by country: Portugal (n = 1), Romania (n = 1), Morocco (n = 4), Cameroon (n = 1), Equatorial Guinea (n = 15), Mozambique (n = 1), Nigeria (n = 4), Argentina (n = 1), Bolivia (n = 2), Colombia (n = 2), Ecuador (n = 4), Guatemala (n = 1), Haiti (n = 1), Honduras (n = 3), Mexico (n = 1), Peru (n = 1), Dominican Republic (n = 1), China (n = 1), and India (n = 1).

^d^Virological features in patients with resistance information. Viral Load: HIV-1 RNA-copies/ml. In bold, significant p values (< 0.05).

### Virological and immunological status of study population with genotypic information

For the present transversal study we included only 133 (72.3%) of 184 transferred and 75 (70.7%) of 106 non-transferred patients of the study cohort with available *pol* sequence or genotypic resistance profiles in their clinical reports. By December 2017 all patients were on ART and most (70.7% of non-transferred and 65.4% of transferred) were virologically suppressed (< 50 RNA cp/ml). Non-transferred patients presented higher median nadir CD4 cells than transferred in percentage (15% vs. 11%; p < 0.0001) and counts (379 [IQR 206–500] vs. 187 [IQR 41.2–345.5]; p < 0.0001), and similar median CD4 percentages (33.3% vs. 31.6%) and CD4 cells counts (781 [IQR 561–962] vs. 725 [IQR 498–901] cells/mm^3^). In both cohorts, around 70% of patients had 25–50% of CD4+ T cells percentage at sampling, and over 70% of them reached > 500 cells/mm^3^ (Table 1). However, statistical differences were observed in CD8 cells measures between both cohorts. Transferred youths showed higher median CD8 percentages (39.6% [IQR 34–53] vs. 36% [IQR 28–42]; p = 0.0004) and counts (873 [IQR 701–1,210] vs. 795 [IQR 552–1,077] cells/mm^3^; p = 0.0232). Nearly half (44%) of pediatric patients achieved CD4/CD8 ratio ≥ 1, whereas a significantly higher number of the transferred cohort had CD4/CD8 ratio < 1 (61.6% vs. 45.3%; p = 0.0291).

At sampling time, most transferred (88%) and non-transferred (66.7%) had previous ARV experience, with higher median age at first ART experience among transferred vs. non-transferred (3.4 [IQR 0.9–6.4] vs. 0.8 [IQR 0.3–4.3] years; p = 0.0023) (Table 2). Thus, ART start occurred significantly earlier after HIV diagnosis in non-transferred than in transferred: 4.5 weeks [IQR 0.4–24.8] vs. 1.5 years [IQR 0.2–4.6] (p < 0.0001). The most common ARV experience among both transferred (43.6%) and non-transferred patients (60%) were mono or dual NRTI-based regimens (Table 2). Double NRTI/NNRTI regimens were significantly less frequent in transferred compared to non-transferred patients (0.9% vs. 8%; p = 0.0285) and triple-class experience including NRTI/NNRTI/PI more frequent (33.3% vs. 18%; p = 0.0612) (Table 2). Experience with other drug families (fusion, integrase, or CCR5 inhibitor) was scarce in both groups. Regarding specific ARVs, stavudina (d4T) exposure was significantly more frequent in transferred (43.6% vs. 20%; p = 0.004) as didanosine (ddI, 44.4% vs. 20%; p = 0.0029) and ritonavir (RTV, 34.2% vs. 6%; p < 0.0001) and abacavir (ABC) were less frequent (12.8% vs. 28%; p = 0.025, data non-shown).

**Table 2:** Virological features and HIV drug resistance mutations in HIV-infected children and transferred with available *pol* sequence or resistance profile at sampling time.

Variable	Non-transferred^a^ (N = 75)	Transferred^b^ (N = 133)	P value
**ART exposure, No. (%)**			
Naïve	23 (30.6)	16 (12.0)	**0.0015**
Treated	50 (66.7)	117 (88.0)	**0.0004**
Unknown	2 (2.7)	0	0.1289
**ART experience, No. (%)**	50	117	
Mono/dual NRTI	30 (60.0)	51 (43.6)	0.8825
NRTI + NNRTI	4 (8.0)	1 (0.9)	**0.0285**
NRTI + PI	3 (6.0)	12 (10.2)	0.5566
Triple (NRTI + NNRTI + PI)	9 (18.0)	39 (33.3)	0.0612
With ≥ 3 family drugs*	1 (2.0)	1 (0.9)	0.5104
Unknown data	3 (6.0)	13 (11.1)	0.3972
**Age at first ART experience, years, median [IQR]**	0.8 [0.3–4.3]	3.4 [0.9–6.4]	**0.0023**
**Time from diagnosis to ART start, median [IQR]**	4.5 weeks [0.4–24.8]	1.5 years [0.2–4.6]	**< 0.0001**
**ART exposure time, years, median [IQR]**	15.2 [10.5–19.4]	22.7 [20.8–24.4]	**< 0.0001**
**Year of last available sequence, No. (%)**			
1993–1997	4 (5.3)	15 (11.3)	0.2112
1998–2002	6 (8.0)	21 (15.8)	0.1342
2003–2007	15 (20.0)	38 (28.6)	0.1888
2008–2012	30 (40.0)	32 (24.1)	**0.0184**
2013–2017	18 (24.0)	27 (20.2)	0.5996
Unknown	2 (2.7)	0	0.1289
**Nº of naïve patients *pol*** **with sequence, No.**	23	16	
**Nº of naïve patients with TDR, No. (%)**	4 (17.4)	2 (12.5)	1.0000
To NRTI	3 (13.0)	2 (12.5)	1.0000
To NNRTI	0	0	
To PI	3 (13.0)	0	0.2550
Double resistance (NRTI + PI)	2 (8.7)	0	0.5033
Triple resistance (NRTI + NNRTI + PI)	0	0	
Only			
To NRTI	1 (4.3)	2 (12.5)	0.5570
To NNRTI	0	0	
To PI	1 (4.3)	0	1.0000
**Nº of pretreated patients, No.**			
With *pol* sequences	49	113	1.0000
With resistance profile	1	4	1.0000
HIV-1 variants prevalence, No. (%)	N = 75	N = 130	
B subtype	51 (68.0)	121 (93.1)	**< 0.0001**
Non-B variants	24 (32.0)	9 (6.9)	**< 0.0001**
Pure non-B subtypes	8 (10.7)	4 (3.1)	**0.0329**
CRF	15 (20.0)	4 (3.1)	**0.0001**
URF	1 (1.3)	1 (0.8)	1.0000
Unknown	0	3 (2.3)	0.3005

*ART* antiretroviral therapy, *NRTI* nucleoside reverse-transcriptase inhibitor, *NNRTI* non-nucleoside reverse-transcriptase inhibitor, *PI* protease inhibitor, *INI* integrase inhibitor, *T20* enfuvirtide, *TDR* transmitted drug resistance, *DRM* drug resistance mutation, *SD* standard deviation, *IQR* interquartile range, *CRF* circulating recombinant form, *URF* unique recombinant form; Subtype information was available in 205 of the 208 patients under study. They included 203 subjects with *pol* sequence and two patients (1 non-transferred and 1 transferred youth) with no available *pol* sequence but available HIV-1 variant information in their clinical report. In bold, significant p values (< 0.05).

*Both transferred adolescent and child had INI, T20 and CXCR5 inhibitor experience.

^a^Patients from Madrid Cohort of HIV-1 infected children and adolescents under follow-up in pediatric units.

^b^Transferred from pediatric to adult units.

### Transmitted resistance among transferred vs. non-transferred patients

Among 208 patients with available resistance profile, 16 (12%) transferred and 23 (31%) non-transferred patients were ART naïve at sampling. Among them, TDR were found in 4 (17.4%) non-transferred and 2 (12.5%) transferred patients based on WHO 2009 SDRM list (Table 2). TDR to NNRTI was more frequent in non-transferred and TDR to NRTI in transferred. TDR mutations found in non-transferred were M41L, D67N, M184V, L210W, T215Y/S in RT and L24I, D30N, V32I, I54V, V82A and N88D in PR. In transferred only M41L in RT.

### High prevalence of HIV-1 resistant variants in pretreated transferred patients to NRTI and PI

**Figure 1:**
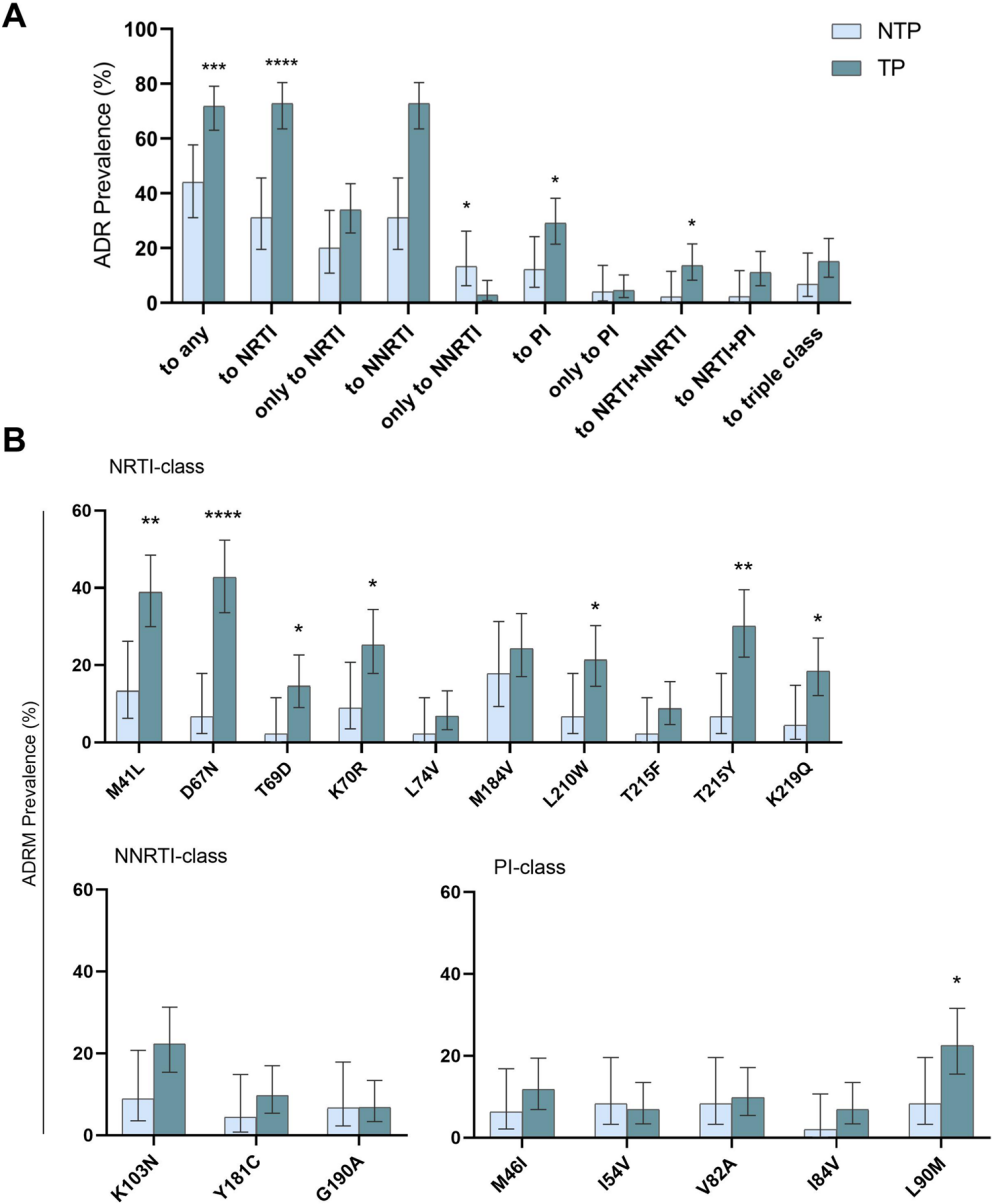
Acquired drug resistant prevalence and the most representative mutations in the study pretreated population from the Madrid Cohort of HIV-1 infected children and adolescents. (**A**) ADR prevalence according to drug class in 167 pretreated patients with *pol* sequence or resistance data. (**B**) ADR prevalence over 5% in 167 pretreated patients. Triple-class: ADR to NNRTI + NRTI + PI. ADR to NNRTI + PI was not found. Error bars indicate exact hybrid Wilson/Brown 95% CIs. Statistical differences: ****p < 0.0001; **p < 0.01; *p < 0.05 Chi-square test. Results were calculated in 49 PR and 45 RT sequences or resistance profiles from non-transferred patients and in 110 PR and 103 RT sequences or resistance profiles from transferred individuals at sampling. *ADR* acquired HIV drug resistance mutations, *NTP* non-transferred patients, *TP* transferred patients.

To assess the acquired drug resistance (ADR) prevalence according to drug family, we analyzed the last available resistance information (*pol* sequence and resistance profile) closest to the end of December 2017 in 50 pediatric and 117 ART-experienced transferred youths (Table 2, Fig. 1a). ADR prevalence was significantly higher in pretreated transferred than non-transferred patients (71.8% vs. 44%; p = 0.0009), mainly to NRTI (72.8% vs. 31.1%; p < 0.0001) and PI (29.1% vs. 12%; p = 0.0262), presenting similar NNRTI resistance (32% vs. 22.2%; p = 0.2453). The presence of triple-class resistant viruses was similar in both groups (15.2% vs. 6.8%; p = 0.2735). Among pretreated patients, we identified these specific ADR present over 5% of treated patients. Seven out ten prevalent ADR to NRTIs in the study population (M41L, D67N, T69D, K70R, L210W, T215Y and K219Q) were significantly more frequent in the transferred vs. the non-transferred cohort, mainly D67N, M41L and T215Y (41.7% vs. 6.7%, p < 0.0001; 38.9% vs. 13.3%, p = 0.0019; 30.1% vs. 6.7%, p = 0.0013, respectively). No significant differences were found in ADR to NNRTI and in ADR to PI L90M, change more frequent in transferred youth (22.6% vs. 8%; p = 0.0405) (Fig. 1b).

**Figure 2:**
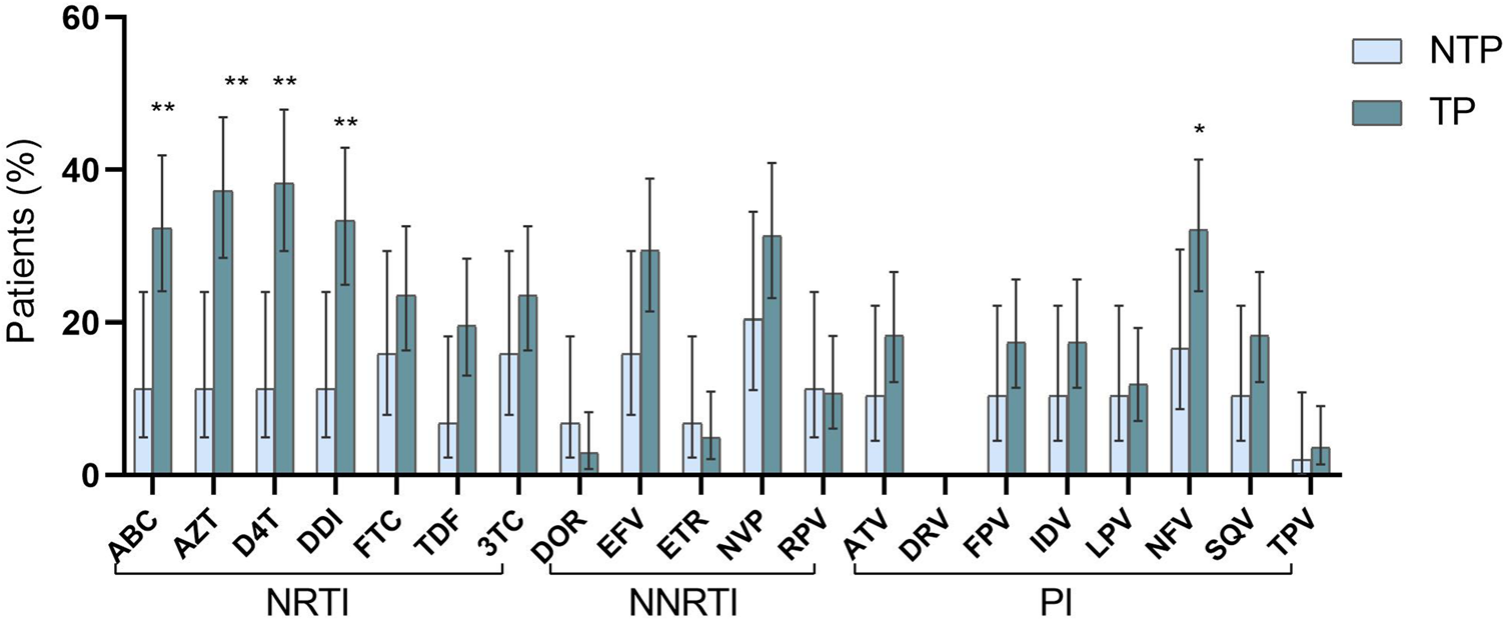
Predicted high resistance level to antiretroviral drugs in pretreated patients from the Madrid Cohort of HIV-1 infected children and adolescents. Susceptibility level was estimated in the 162 pretreated patients with available *pol* sequence according to the Stanford HIVdb Interpretation Algorithm. Error bars indicate exact hybrid Wilson/Brown 95% CIs. Statistical differences: **p < 0.01 Chi-square test. Results were calculated in 48 PR and 44 RT sequences from non-transferred patients and in 109 PR and 102 RT sequences from transferred individuals at sampling. *ABC* abacavir, *AZT* zidovudine, *d4T* stavudine, *ddI* didanosine, *FTC* emtricitabine, *TDF* tenofovir disoproxil fumarate, *3TC* lamivudine, *DOR* doravirine, *EFV* efavirenz, *ETR* etravirine, *NVP* nevirapine, *RPV* rilpivirine, *ATV* atazanavir, *DRV* darunavir, *FPV* fosamprenavir, *IDV* indinavir, *LPV* lopinavir, *NFV* nelfinavir, *SQV* saquinavir, *TPV* tipranavir, *NRTI* nucleoside reverse-transcriptase inhibitor, *NNRTI* non-nucleoside reverse-transcriptase inhibitor, *PI* protease inhibitor.

Figure 2 shows the predicted resistance level to 20 ARV of the most used drug families (NRTI, NNRTI, PI) among the 162 pretreated subjects under study carrying ADR (49 non-transferred and 113 transferred) with available *pol* sequences. The transferred cohort reported a significantly higher rate of patients with high resistance level to a NRTI family drugs than non-transferred (60.1% vs. 27.2%; p = 0.0081), mainly to d4T (38.2% vs. 11.4%; p = 0.001), AZT (37.3% vs. 11.4%; p = 0.002), ddI (33.3% vs. 11.4%; p = 0.006) and ABC (32.4% vs. 11.4%; p = 0.008). The rate of non-transferred and transferred patients with predicted high resistance level to NNRTI and PI did not show significant differences, except for nelfinavir (NFV), with higher rates of resistance among transferred youths.

By contrast, a significant highest rate of non-transferred patients were susceptible to all NRTI, NNRTI and PI drugs compare to transferred youths (70.4% vs. 28.4%, p < 0.0001; 61.4% vs. 32.3%, p = 0.0011 and 83.3% vs. 58.4%, p = 0.0011, respectively). Additional complementary data relating to low and intermediate drug resistance levels for each drug in the study population are reported in Supplementary Fig. S1. We identified the specific drugs with the highest susceptibility in both cohorts, representing interesting alternatives for rescue ART regimens if required. Most PI and the new NNRTI (DOR, RPV, and ETR) were the drugs showing the highest susceptibilities in transferred youths.

### HIV-1 variants prevalence in the study population

HIV-1 variant was known in 205 (98.6%) of the 208 patients with available *pol* sequence (n = 203) genotypic resistance profile (n = 5). Among them, 203 (97.6%) could be successfully subtyped by phy. Overall, 172 (83.9%) of the 205 HIV-1 subjects were infected with HIV-1 subtype B^*pol*^, being the predominant variant, as previously reported^9,10^. HIV-1 subtype B infections were more frequent than by non-B variants (83.9% vs. 16.1%; p < 0.0001) in the whole cohort.

Nevertheless, subtype B^*pol*^ prevalence was significantly higher among transferred vs. pediatric cohort (93.1% vs. 68%; p < 0.0001). Non-transferred patients presented a higher prevalence of pure non-B variants at *pol* (10.7% vs. 3.1%; p = 0.033) and inter-subtype recombinant (CRF and URF) variants than transferred (21.3% vs. 3.9%; p = 0.0001).

Among the 33 patients (24 non-transferred and 9 transferred) infected by HIV-1 non-B variants at *pol*, all but 5 cases were born abroad or at least one of their parents were immigrants coming from Sub-Saharan Africa, Eastern Europe or Latin America (Supplementary Table S1). Among the 24 non-transferred patients carrying non-B variants, 6 (25%) were pure non-B subtypes (3C, 1A, 1A6, 1F1), 17 (70.8%) were CRF, mainly CRF02_AG (8 cases, 33.3%) and CRF01_AE (2 cases, 8.3%), the most globally distributed CRFs. We also found URF (1 case, 4.2%) from Equatorial Guinea. Among the 9 transferred youths carrying non-B variants at *pol,* 3 (33.3%) were infected by pure non-B variants (1A, 1A2, 1H), 4 (44.4%) were CRF (1 CRF01_AE, 1 CRF02_AG, 1 CRF12_BF, and 1 CRF28_BF) and born in Spain, and the remaining 2 carried URF including subtype C or G sequences, respectively (Supplementary Table S1).

## Discussion

Transition to adult care is crucial for HIV-infected adolescents. This population faces with important challenges to ensure long-term virological suppression when reaching adulthood. However, little is known about their current health status in each country, despite an expected increase in the number of children being transferred into adult units in the coming years. Several studies have assessed the current state of adolescent survivors of perinatally or early acquired HIV^9, 11–17^. Table 3 shows all related studies on HIV-1 patients transferred from paediatric to adult units worldwide.

**Table 3:** Comparison of published studies from HIV-1 patients transferred from pediatric care to adult units worldwide.

Country^(ref)^	No.	Date	Perinatal infection	Median age (years)			ART experience (mean, year)	Rate of DRM in pre-treated patients						Most frequent DRM	TDR in naïve patients	HIV non-B variants	Clinical status	
				At transfer	HIV DX	First cART		No of polseq	To any	Only to NRTI	Only to NNRTI	Only to PI	Dual^a^/Triple^b^				CD4 > 500 cells/mm^3^	UVL
Present study	184	1997–2017	94%	18.7	1.3	3.5	22.7	113	75.2%	23.1%	2.6%	7%	28.2%/15.4%	NRTI: D67N NNRTI: K103N PI: L90M	19%	6.9%	74%	65.4%
Spain^9^	112	1997–2011	93.7%	18.9	2	5.6	11.5	58	81%	28.5%	7.1%	14.6%	31%/17.3%	NRTI: M41L NNRTI: K103N PI: L90M	0%	1.9%	55.3%	38.4%
UK/Ireland^11^	644	1996–2016	91%	17.4	6.4	9.6	7.8	381	82%	9.3%	16.2%	0.7%	44%/12%	NRTI: M184V NNRTI: K103N PI: L90M	6%	–	42%	60%
Sweden^12^	34	2013–2015	91%	19	–	9	–	32	–	–	–	–	25%/–	–	–	–	–	96%
The Netherlands^13^	54	1996–2014	78%	18.8	8.4	10.4	–	–	–	–	–	–	–	–	–	–	–	–
Italy^14^	24	2004–2006	100%	18	–	–	14	13	69.2%	–	–	–	46.2%/–	NRTI: M41L	–	–	–	75%
USA^15^	735	2006–2015	100%	22	–	–	–	–	–	–	–	–	–	–	–	–	38.9%	51.8%
Canada^16^	45	1999–2011	71%	18.1	–	–	–	38	73.7%	21%	55.3%	50%	–/31.6%	–	–	20%	28.9% < 200 cells/ml	42.2%
Argentina^17^	37	2011	100%	18	–	–	15	–	–	–	–	–	–/45%	NRTI: D67N NNRTI: K103N PI: V82A	–	–	36.3%	45%

*HIV* human immunodeficiency virus, *ref* reference, *No.* number of transferred participants in each study, *DX* diagnosis, *cART* combination antiretroviral therapy, *ART* antiretroviral therapy, *seq* sequences, *DRM* drug resistance mutation, *NRTI* nucleoside reverse-transcriptase inhibitor, *NNRTI* non-nucleoside reverse-transcriptase inhibitor, *PI* protease inhibitor, *UVL* undetectable viral load ≤ 50 RNA copies/ml at last available viraemia, except from USA study^23^ (< 400 cp/ml), *cp* copies; dash: not provided data. HIV non-B variants include HIV-1 subtypes different than subtype B and recombinants.

^a^DRM found to NRTI + NNRTI, NRTI + PI and NNRTI + PI drug class families.

^b^DRM found to NRTI + NNRTI + PI drug class families.

During the early nineties, Spain had the highest incidence of mother-to-child transmission in Western Europe among heroin users HIV-infected women, leading to high HIV transmissions in children born between 1980 and 1990^18^. The Madrid cohort is one of the best characterized perinatal cohort in Europe and worldwide, along with the UK/Ireland^11^, the Netherlands^13^ and New York City^15^ cohorts (Table 3). Future transitioning programs will represent a challenge mainly in low-income countries^28,29^ where most HIV-infected-children and adolescents live^19,31^.

By the end of December 2017, two-thirds of the perinatally infected patients in our cohort in Madrid (Spain) had reached adolescence and transitioned to adult care. Here, despite similar median age at transfer, transferred youths in the present study were younger at HIV diagnosis (1.3* vs*. 2 years old) and at first ART experience (3.4 vs. 5.6 years old) than the same cohort 6 years before^9^. Additionally, over six years, our perinatal cohort had improved their immunological status significantly regarding the rate of transferred achieving CD4 T cells > 500 cells/mm^3^ counts (74% vs. 55.3% reported in 2011; p = 0.0031), reaching rates higher than in comparable studies in UK/Ireland^11^ (42%), New York^15^ (38.9%) and Argentina^17^ (36.3%). The good recovery of CD4 counts in the Madrid cohort could be due to an early diagnosis and treatment and improved ART regimens, in agreement with other studies reporting that better initial status is associated with improved immune recovery^20–22^.

Regarding virological outcome, our updated data showed a 27% increase in transferred youths with available sequence with undetectable viral load (UVL) in our Spanish cohort from 2011 to 2017 (38.4% vs. 65.4%; p < 0.0001) (Table 3). Swedish and Italian cohorts presented higher rates of transferred patients achieving UVL^12,14^ and Canadian and Argentina transferred cohorts the lowest^16,17^ (42.2% vs. 45%, respectively), despite being considered high-income countries. Nevertheless, in some high-income countries, transferred young people still have high rates of virological failure immediately before, during, and shortly after transition (36% in the Netherlands)^13^, as well as a loss of follow-up after transition (nearly 14% in Spain^23^ and in the Netherlands^13^), mainly in the first year after transfer.

By the end of December 2017, a third of transferred youths still had incomplete viral suppression, and lower median CD4/CD8 ratio than non-transferred patients, a predictor of increased immunoactivation and immunosenescence despite ART^24^. The incomplete viraemia suppression could be explained by partial adherence to treatment, a key problem in adolescence. Moreover, most transferred youths of the study cohort was infected in the mono and bi-therapy era, receiving several suboptimal treatments and selecting a high rate of historic DRM, one of the major obstacles for an effective ART^25^. The complex clinical management in perinatally-infected youths impacts in the current immune-virological control of HIV infection compared to adults and to patients under pediatric care, who probably have received optimal ART regimens. Thus, better immune-virological situation during transition to adult units is expected in future transferred cohorts.

We observed a high TDR rate in the study cohort, mainly in the transferred group. The higher prevalence of resistance found in transferred vs. non transferred individuals could be due to the older age and longer therapy experience with less efficacious antiretroviral treatments and many regimen switches vs. non-transferred. The transferred adolescents had to face the monotherapy and dual therapy regimens available at the time, thus increasing the risk of virological failures and unsuppressed viraemia due to resistance development. In our study, the 4 transferred with TDR were vertically HIV-1 infected adolescents, collective found to have higher risk of treatment failure than newly HIV infected youth, probably as a result of their lifelong infection and their heavily ART exposition^6^. Moreover, HIV infected patients during childhood in our transferred cohort were mainly infected during the early 1990s, when Spain had one of the highest rates of AIDS in Europe. The inadequate ART regimens in their HIV-infected mothers could also contribute to the high resistant level found in perinatally infected transferred group.

The ART expansion in low-income countries where most pediatric infections occur and the insufficient adherence support, frequent suboptimal ART regimens in HIV-infected mothers and children, lack of routine viral load (VL) and resistance monitoring in most of these settings, can lead to the spreading of resistant viruses among new infections in naïve and treated children. In fact, nearly 85% of naïve non-transferred patients in our study were born abroad or from HIV-infected parents coming from low-income countries, where ART has been expanding in the last years, without the implementation or availability of optimal ART regimens^26^. TDR rate in perinatally HIV-1 infected patients in Madrid was higher than in perinatal cohorts from UK/Ireland (6%)^11, 27^, and in most pediatric cohorts worldwide^28^, as well as in adults from Europe (8.3%)^29^ and in the Spanish AIDS Research Network of adults (7.9%)^30^. The presence of TDR has important clinical consequences due to the influence of baseline drug resistance patterns in the outcome of first-line ART in children^31^ and adults^29^ and is a strong predictor of treatment failure.

ADR prevalence among ART-experienced patients has decreased over time in the Madrid cohort for all drug families (Supplementary Table S2). The significant reduction in the rate of non-transferred patients with ADR to NRTI class (from 62.1% in 2011 to 28% in 2017; p < 0.0001) could likely be due to the implementation of LPV/r as a first-line combined antiretroviral treatment (cART) in Spain since 2008, and the withdrawal of NFV in 2007^32^. The significant decrease for ADR to PI in patients under pediatric care and transferred youths in Spain could reflect the improvements in ART due to availability of new drug classes in the last years. Nevertheless, in 2017 transferred patients still maintained the highest ADR prevalence to NRTI (64.1%), since it was the first available drug class for clinical use, ABC and AZT being the most compromised drugs, along with ddI and d4T no-longer-used ARV comparing to non-transferred patients (Fig. 2). This was due to the higher presence of D67N, M41L and T215Y resistance mutations in RT in 42.7%, 38.8% and 30.1% of transferred youth, respectively.

Triple-class resistance was detected in 15.2% of transferred youths, a lower rate than the one previously reported in the same study cohort (17.3%), as in other transferred cohorts in Canada (31.6%)^16^ and Argentina (45%)^17^, and higher than in UK (12%)^11^. All patients carrying triple-class resistance in our cohort were born between 1987 and 1996, and 43.9% of them had experienced mono/dual NRTI therapies before cART implementation, which may have led to treatment failure and subsequent ADRM selection due to the incomplete viral suppression^33^. It is important to highlight that the comparison between transferred and non-transferred patients was completely related with the time-period when they were infected and treated, suggesting that a direct comparison may not be accurate in this study.

Despite high ADR rate to the three main ARV families among transferred, our data showed that some NNRTI (DOR, ETR, and RPV) and PI (DRV and TPV) remained good options to rescue the highly pretreated patients in the study cohort. Moreover, nowadays young people could benefit from newly licensed drugs to treat HIV-1 in adults, like cell-entry and integrase inhibitors^34^. Surveillance of TDR and ADR prevalence among HIV-1 infected children and adolescents is critically important in determining if changes to empiric first, second and third-line ART regimens are required^35^.

Regarding HIV infecting variants, infections with non-B variants in non-transferred patients increased significantly from 2011 to 2017 (11.5% to 32%; p = 0.0004), mostly due to the increment of children infected by CRF (6.9% vs. 20%; p = 0.0065). Despite that fact that subtype B was the prevalent variant in the transferred cohort, non-B infections also increased among transferred from 1.9% (2011) to 6.9% (2017) (Table 3). Inevitably, this viral heterogeneity could affect the efficacy of HIV-1 monitoring, affecting the clinical management of HIV-1 infection or disease progression^36,37^.

The main limitation of the study is that resistance results derived from available *pol* sequence or resistance profiles closest to December 2017, ranging from 1993 to 2017 but mainly dating from 2005 to 2010 (Table 2). Therefore, resistance patterns may not precisely reflect features in December 2017. Moreover, we only used data from patients with available resistance testing, excluding of the virological study patients without *pol* sequences. VL quantification assays with different limit of detections differed across patients and years during the clinical follow-up of the study cohort.

The world is home to more young people (ages 10–24 years old) now than at any other time in history, and we need to focus and care for this collective if we want to end the AIDS epidemic by 2030. Our study demonstrated that good clinical management could achieve the goal that most HIV-1-infected patients transferred from pediatric care to adult units may maintain virological suppression and high CD4 counts, decreasing ADR prevalence and improving their clinical status. This highlights the importance of VL and drug resistance monitoring worldwide in all HIV-infected-pediatric and young population for ART optimization if required during the chronic clinical follow-up of infection.

## Methods

### Study population

In this multicenter observational retrospective and transversal study, we identified 290 patients enrolled in the Madrid Cohort of HIV Infected Children and Adolescents including all youths transferred from pediatric care to adult units and all non-transferred patients by December 2017. Among them, 208 (133 transferred and 75 non-transferred patients) had at least one available HIV-1 polimerase (*pol*) sequence or genotypic resistance profiles in their clinical reports, presenting similar demographical and clinical features to the overall population. For resistance testing and HIV-1 variant characterization we selected the sequence/profile closest to December 2017 per patient, defining as sampling time the year of collection of sequenced samples, which ranged from 1993 to 2017. Most sequences were previously reported by our group^9,10^, except 20 new *pol* genotypes recovered from hospitals. We also collected retrospective epidemiological-virological data from clinical records closest to December 2017: origin, gender, age, HIV transmission route, HIV diagnosis date, antiretroviral therapy (ART) experience, CD4 and CD8 counts (percentage and cells/mm^3^), CD4/CD8 rate, and viral load (HIV- 1 RNA copies/ml of plasma, cp/ml). This study was approved by the Clinical Research Ethical Committee at University Hospital Ramón y Cajal (Madrid, Spain). All methods were carried out in accordance with relevant guidelines and regulations. Informed consent was obtained from all subjects or, if subjects are under 18, from a parent and/or legal guardian.

### Drug resistance analysis

The acquired HIV drug resistance mutations (ADR) in pretreated patients to nucleoside reverse-transcriptase inhibitors (NRTIs), non-nucleoside reverse-transcriptase inhibitors (NNRTIs) and major protease inhibitors (PI) were defined by the HIVdb Program Genotypic Resistance Interpretation Algorithm v8.9-1 (Stanford University, Palo Alto, CA, USA)^38^. In drug-naïve patients, the prevalence of transmitted drug resistance mutations (TDR) was established according to the mutation list as recommended by the WHO^39^ and using the Calibrated Population Resistance tool v8.0^40^. Drug susceptibility was predicted for 20 available antiretroviral drugs inhibitors according to Stanford.

### HIV-1 subtyping

DNA sequences were aligned using Muscle tool in MEGAv6.0.6 and phylogenetic analysis (phy) for subtyping was performed using Maximum likelihood and General Time Reversible as the evolutionary model with 1,000 bootstrap resampling. The bootstrap cut-off was set at 70. For phy construction, we used as references at least 2 representative *pol* sequences from each group M variant (9 subtypes, 6 sub-subtypes and 76 of 98 described HIV circulating recombinant forms [CRF]) with available sequences at GenBank at the time of the analysis. Sequences not identified as any known group M subtype, sub-subtype or CRF by phy were considered HIV-1 group M unique recombinant forms (URF) in *pol* (URF^*pol*^).

### Statistical analysis

To compare the pediatric and transferred cohorts the Fisher exact test and Chi-square test were used for categorical variables. The unpaired Student t test or the Mann–Whitney test was performed for continuous variables. Means and standard deviations (SD) were used for normally distributed data, and medians and interquartile ranges (IQR) for data that are not normally distributed. To compute the 95% confidence interval (95% CI) we use the hybrid Wilson/Brown method for the sensitivity/specificity and the Newcombe/Wilson method to calculate the difference between proportions. All analyses were performed by using GraphPad Prism 8.0.1. Two-sided p-values of < 0.05 were considered statistically significant.

## Supplementary information

Supplementary Information.

## References

[1] UNAIDS data 2019|UNAIDS. https://www.unaids.org/en/resources/documents/2019/2019-UNAIDS-data.

[2] HIV and AIDS in Adolescents—UNICEF Data. https://data.unicef.org/topic/adolescents/hiv-aids/.

[3] Flynn PM, Abrams EJ (2019). Growing up with perinatal HIV. AIDS.

[4] Rojas Sánchez P (2015). Clinical and virologic follow-up in perinatally HIV-1-infected children and adolescents in Madrid with triple-class antiretroviral drug-resistant viruses. Clin. Microbiol. Infect..

[5] Fish R, Judd A, Jungmann E, O’Leary C, Foster C (2014). Mortality in perinatally HIV-infected young people in England following transition to adult care: an HIV Young Persons Network (HYPNet) audit. HIV Med..

[6] Judd A (2017). Higher rates of triple-class virological failure in perinatally HIV-infected teenagers compared with heterosexually infected young adults in Europe. HIV Med..

[7] Judd A (2017). Growing up with perinatal HIV: changes in clinical outcomes before and after transfer to adult care in the UK. J. Int. AIDS Soc..

[8] WHO | Global action plan on HIV drug resistance 2017–2021. *WHO* (2017).

[9] de Mulder M (2012). High drug resistance prevalence among vertically HIV-infected patients transferred from pediatric care to adult units in Spain. PLoS ONE.

[10] Rojas Sánchez P (2018). Trends in drug resistance prevalence, HIV-1 variants and clinical status in HIV-1-infected pediatric population in Madrid: 1993 to 2015 analysis. Pediatr. Infect. Dis. J..

[11] Collins IJ (2017). Clinical status of adolescents with perinatal HIV at transfer to adult care in the UK/Ireland. Clin. Infect. Dis..

[12] Westling K, Navér L, Vesterbacka J, Belfrage E (2016). Transition of HIV-infected youths from paediatric to adult care, a Swedish single-centre experience. Infect. Dis. (Auckl).

[13] Weijsenfeld AM (2016). Virological and social outcomes of HIV-infected adolescents and young adults in The Netherlands before and after transition to adult care. Clin. Infect. Dis..

[14] Izzo I (2018). Perinatally HIV-infected youths after transition from pediatric to adult care, a single-center experience from Northern Italy. AIDS Res. Hum. Retroviruses.

[15] Xia Q (2018). Transition from paediatric to adult care among persons with perinatal HIV infection in New York City, 2006–2015. AIDS.

[16] Van der Linden D (2013). The young and the resistant: HIV-infected adolescents at the time of transfer to adult care. J. Pediatr. Infect. Dis. Soc..

[17] Córdova E, Yañez J, Rodriguez Ismael CG (2015). Safety and efficacy of antiretroviral therapy in perinatally HIV-1 infected patients following transition to an adult HIV-care hospital with virological failure in Buenos Aires, Argentina. Enferm. Infecc. Microbiol. Clin..

[18] Palladino C (2008). Spatial pattern of HIV-1 mother-to-child-transmission in Madrid (Spain) from 1980 till now: Demographic and socioeconomic factors. AIDS.

[19] 2015 Annual Results Reports | UNICEF Publications | UNICEF. https://www.unicef.org/publications/index_91618.html.

[20] Puthanakit T (2009). Pattern and predictors of immunologic recovery in human immunodeficiency virus-infected children receiving non-nucleoside reverse transcriptase inhibitor-based highly active antiretroviral therapy. Pediatr. Infect. Dis. J..

[21] Kekitiinwa A (2008). Differences in factors associated with initial growth, CD4, and viral load responses to ART in HIV-infected children in Kampala, Uganda, and the United Kingdom/Ireland. J. Acquir. Immune Defic. Syndr..

[22] Patel K (2008). Long-term effects of highly active antiretroviral therapy on CD4^+^ cell evolution among children and adolescents infected with HIV: 5 Years and counting. Clin. Infect. Dis..

[23] Sainz T, Navarro ML (2017). HIV-infected youths: Transition in Spain compared to the Netherlands. Clin. Infect. Dis..

[24] Sainz T (2013). The CD4/CD8 ratio as a marker T-cell activation, senescence and activation/exhaustion in treated HIV-infected children and young adults. AIDS.

[25] Ammaranond P, Sanguansittianan S (2012). Mechanism of HIV antiretroviral drugs progress toward drug resistance. Fundam. Clin. Pharmacol..

[26] Yebra G (2011). Increase of transmitted drug resistance among HIV-infected sub-Saharan Africans residing in Spain in contrast to the native population. PLoS ONE.

[27] Chakraborty R (2008). HIV-1 drug resistance in HIV-1-infected children in the United Kingdom from 1998 to 2004. Pediatr. Infect. Dis. J..

[28] Rojas Sánchez P, Holguín A (2014). Drug resistance in the HIV-1-infected paediatric population worldwide: a systematic review. J. Antimicrob. Chemother..

[29] Hofstra LM (2016). Transmission of HIV drug resistance and the predicted effect on current first-line regimens in Europe. Clin. Infect. Dis..

[30] Monge S (2014). Clinically relevant transmitted drug resistance to first line antiretroviral drugs and implications for recommendations. PLoS ONE.

[31] Wittkop L (2011). Effect of transmitted drug resistance on virological and immunological response to initial combination antiretroviral therapy for HIV (EuroCoord-CHAIN joint project): a European multicohort study. Lancet Infect. Dis..

[32] Rojas Sánchez P (2017). Impact of lopinavir–ritonavir exposure in HIV-1 infected children and adolescents in Madrid, Spain during 2000–2014. PLoS ONE.

[33] Englund JA (1997). Zidovudine, didanosine, or both as the initial treatment for symptomatic HIV-infected children. N. Engl. J. Med..

[34] Clinical guidelines: antiretroviral therapy 4.1 Preparing people living with HIV for ART. https://www.who.int/hiv/pub/arv/chapter4.pdf?ua=1.

[35] Nuttall J, Pillay V (2019). Antiretroviral resistance patterns in children with HIV infection. Curr. Infect. Dis. Rep..

[36] Santoro MM, Perno CF (2013). HIV-1 genetic variability and clinical implications. ISRN Microbiol..

[37] Bhargava M, Cajas JM, Wainberg MA, Klein MB, Pai NP (2014). Do HIV-1 non-B subtypes differentially impact resistance mutations and clinical disease progression in treated populations? Evidence from a systematic review. J. Int. AIDS Soc..

[38] HIVdb Program: Sequence Analysis—HIV Drug Resistance Database. https://hivdb.stanford.edu/hivdb/by-sequences/.

[39] Bennett DE (2009). Drug resistance mutations for surveillance of transmitted HIV-1 drug-resistance: 2009 update. PLoS ONE.

[40] CPR: Calibrated Population Resistance Tool. https://cpr.stanford.edu/cpr.cgi.

