# Supplementary material for: Virological outcome among HIV infected patients transferred from pediatric care to adult units in Madrid, Spain (1997–2017)
Source: Sci Rep. 2020 Oct 9;10:16891. doi: 10.1038/s41598-020-70861-x (PMC7547007; doi:10.1038/s41598-020-70861-x)
Supplement: Supplementary file 1 — Supplementary Information. [file 41598_2020_70861_MOESM1_ESM.pdf]

SUPLEMENTARY MATERIAL

- Supplementary Table S1. HIV-1 non-B variants characterized by phylogenetic analysis of *pol* sequences in the study Madrid Cohort of HIV-1 infected children and adolescents.
- Supplementary Table S2. Trends in ADR prevalence in pretreated non-transferred and transferred youths in the Madrid Cohort of HIV-1 infected children and adolescents (2011 *vs.* 2017).
- Supplementary Fig. S1. Predicted susceptibility level to antiretroviral drugs in pretreated patients from the Madrid Cohort of HIV-1 infected children and adolescents.

Supplementary Table S1. HIV-1 non-B variants characterized by phylogenetic analysis of *pol* sequences in the study Madrid Cohort of HIV-1 infected children and adolescents.

Cohort	GeneBank Access number	Available sequence	HIV-1 variant	Infection route	Place of birth (Patient)	Father origin	Mother origin
**Non-transferred patients (N=24)**	MT085808	PR+RT	A	Unknkown	EQ	-	-
	JQ351963	PR+RT	A6	Perinatal	Spain	Bulgaria	Ukraine
	JQ351964	PR+RT	C	Perinatal	Mozambique	-	-
	JQ351994	PR+RT	C	Perinatal	Morocco	-	-
	HQ426895	PR+RT	C	Perinatal	Spain	Cameroon	Spain
	KX607065	PR+RT	CRF11_cpx	Perinatal	Spain	EQ	EQ
	KX607074	PR+RT	CRF_012BF	Perinatal	Spain	Spain	Paraguay
	MT085809	PR+RT	CRF01_AE	Sexual	Bolivia	-	-
	genotypic resistance profile available[a]		CRF01_AE	Perinatal	EQ	-	-
	JQ351975	PR+RT	CRF02_AG	Perinatal	Spain	EQ	Spain
	MT085810	PR+RT	CRF02_AG	Perinatal	Nigeria	-	-
	JQ352008	PR+RT	CRF02_AG	Perinatal	Spain	Cameroon	Cameroon
	KX607071	PR+RT	CRF02_AG	Perinatal	Nigeria	-	-
	KX607072	PR+RT	CRF02_AG	Perinatal	EQ	-	-
	HQ426906	PR+RT	CRF02_AG	Perinatal	EQ	-	-
	MT085811	PR+RT	CRF63_2A	Perinatal	Spain	Nigeria	EQ
	HQ426905	PR+RT	CRF02_AG	Perinatal	EQ	-	-
	JQ351971	PR+RT	CRF02_AG	Perinatal	Spain	EQ	EQ
	JQ351953	PR+RT	CRF13_cpx	Transfusion	EQ	-	-
	HQ426911	PR+RT	CRF13_cpx	Perinatal	India	-	-
	MT085812	PR+RT	CRF45_cpx	Perinatal	EQ	-	-
	KX607062	PR+RT	F1	Perinatal	Spain	Romania	Romania
	MT085813	PR+RT	CRF43_02G	Perinatal	Nigeria	-	-
	KX607078	PR+RT	URF con G	Unknkown	EQ	-	-
**Transferred youths (N=9)**	genotypic resistance profile available[a]		A	Sexual	Spain	Spain	Spain
	HQ426893	PR+RT	A2	Perinatal	Spain	Spain	Spain
	MT085814	PR+RT	CRF01_AE	Sexual	Spain	Spain	Spain
	HQ426903	PR+RT	CRF02_AG	Perinatal	Spain	Spain	Morocco
	HQ426910	PR+RT	CRF12_BF	Perinatal	Spain	Spain	Spain
	HQ426759	PR+RT	CRF28_BF	Perinatal	Spain	Spain	Spain
	JQ351962	PR+RT	URF with G	Perinatal	Nigeria	-	-
	HQ426900	PR+RT	H	Perinatal	Cameroon	-	-
	MT085815	PR+RT	URF with B	Sexual	Dominican Republic	-	-

PR, protease; RT, reverse-transcriptase; dash, not available data; EQ, Equatorial Guinea.

In 1 children and 1 transferred with no available *pol* sequence, HIV-1 variant information was available in clinical report.

[a]Genotypic resistance profile: no available sequence.

Supplementary Table S2. Trends in ADR prevalence in pretreated non-transferred and transferred youths in the Madrid Cohort of HIV-1 infected children and adolescents (2011 *vs*. 2017).

ADRM	Pretreated Non-transferred patients. No. (%)			Pretreated transferred youths No. (%)		
	**2011[a] n=131**	**2017[b] n=50**	**p value**	**2011[a] n=58**	**2017[b] n=117**	**p value**
**To any drug family**	91 (69.5)	22 (44)	**0.0016**	47 (81)	84 (71.8)	0.1848
**To NRTI**	81 (62.1)	14 (28)	**<0.0001**	45 (76.9)	75 (64.1)	0.0705
**To NNRTI**	53 (40.5)	10 (20)	**0.0098**	21 (36.5)	33 (28.2)	0.2807
**To PI**	48 (36.8)	6 (44)	**0.0012**	30 (50.9)	32 (27.4)	**0.0015**
**To NRTI+NNRTI**	16 (12.2)	1 (2.0)	**0.0352**	7 (12.1)	14 (10.3)	0.9842
**To NRTI+PI**	11 (8.4)	1 (2.0)	0.1219	11 (19.0)	11 (9.4)	0.0724
**To NNRTI+PI**	1 (0.8)	0	0.5356	0	0	
**To NRTI+NNRTI+PI**	23 (17.6)	3 (6.0)	**0.0474**	10 (17.2)	15 (12.8)	0.4314

No., number of patients. Significant ($p<0.05$).

[a]Previous study: Mulder *et al*, 2012[13]; [b] Present study.

Supplementary Fig. S1. Predicted susceptibility level to antiretroviral drugs in pretreated patients from the Madrid Cohort of HIV-1 infected children and adolescents.

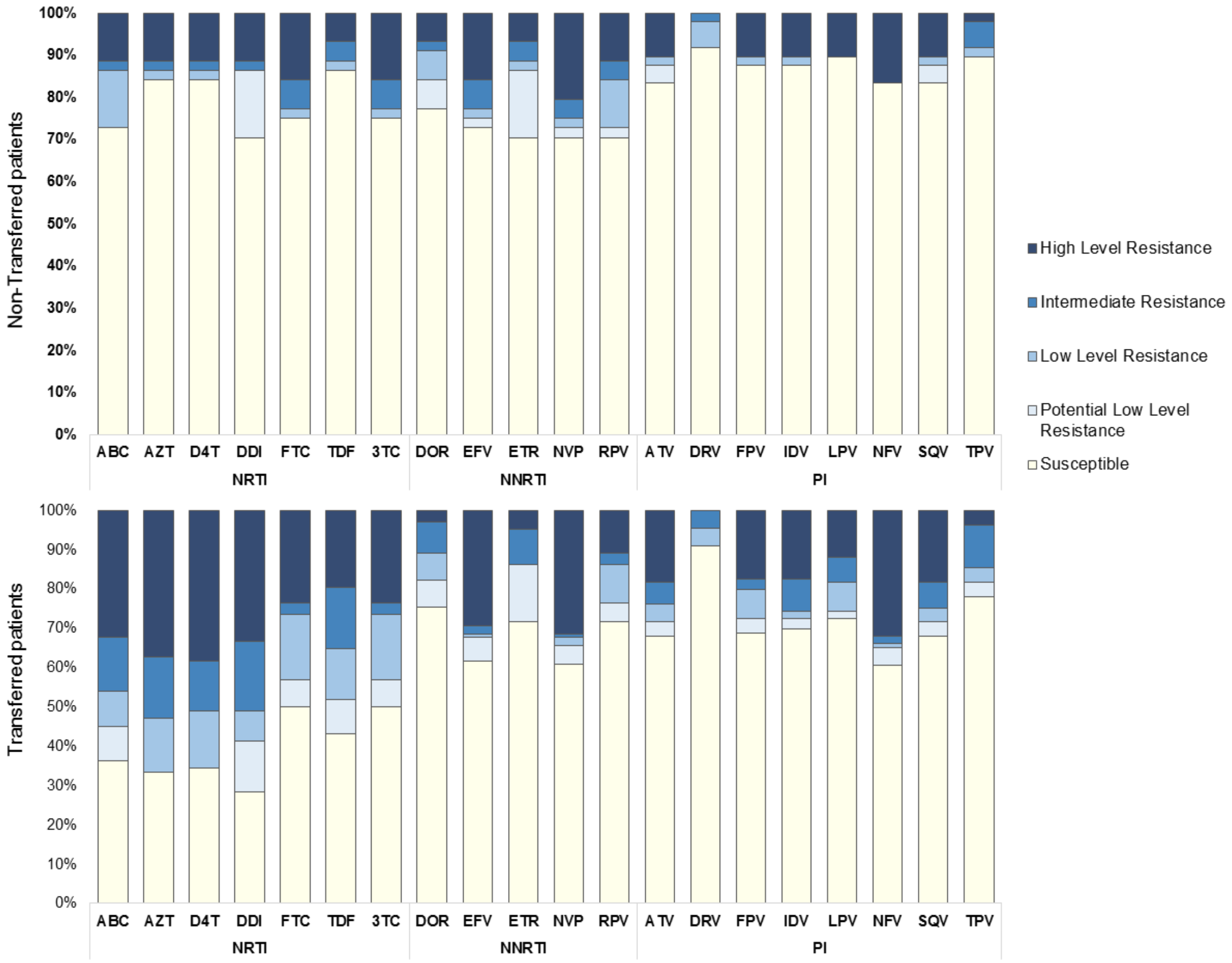

Susceptibility level was estimated according to Stanford HIVdb Interpretation Algorithm.

ABC, abacavir; AZT, zidovudine; d4T, stavudine; ddI, didanosine; FTC, emtricitabine; TDF, tenofovir disoproxil fumarate; 3TC, lamivudine; DOR, doravirine; EFV, efavirenz; ETR, etravirine; NVP, nevirapine; RPV, rilpivirine; ATV, atazanavir; DRV, darunavir; FPV, fosamprenavir; IDV, indinavir; LPV, lopinavir; NFV, nelfinavir; SQV, saquinavir; TPV, tipranavir; NRTI, nucleoside reverse-transcriptase inhibitor; NNRTI, non-nucleoside reverse-transcriptase inhibitor; PI, protease inhibitor.

Results were calculated in 48 PR and 44 RT sequences from non-transferred patients and in 109 PR and 102 RT sequences from transferred individuals at sampling.
